# Use of Propolis Hydroalcoholic Extract to Treat Colitis Experimentally Induced in Rats by 2,4,6-Trinitrobenzenesulfonic Acid

**DOI:** 10.1155/2013/853976

**Published:** 2013-09-11

**Authors:** Cely Cristina Martins Gonçalves, Luzmarina Hernandes, Ciomar Aparecida Bersani-Amado, Selma Lucy Franco, Joaquim Felipe de Souza Silva, Maria Raquel Marçal Natali

**Affiliations:** ^1^Laboratory of Animal Histology, Department of Morphological Sciences, State University of Maringá, 87020-900 Maringá, PR, Brazil; ^2^Laboratory of Inflammation, Department of Pharmacology and Therapeutics, State University of Maringá, 87020-900 Maringá, PR, Brazil; ^3^Laboratory of Phytotherapy and Apitherapy Development, Department of Pharmacy, State University of Maringá, 87020-900 Maringá, PR, Brazil

## Abstract

This study focused on the therapeutic effect of a propolis SLNC 106^*PI*^ extract on experimental colitis. Wistar adult rats received 0.8 mL rectal dose of one of the following solutions: saline (group S), 20 mg TNBS in 50% ethanol (group TNBS), 20 mg TNBS in 50% ethanol and propolis extract in saline (group TNBS-P), propolis extract in saline (group SP), and 20 mg TNBS in 50% ethanol and 50 mg/kg mesalazine (group TNBS-M). The animals were euthanized 7 or 14 days after the colitis induction. Samples of the distal colon were harvested for the analysis of myeloperoxidase (MPO) enzyme activity and for morphometric analysis in paraffin-embedded histological sections with hematoxylin-eosin or histochemical staining. The animals treated with TNBS exhibited the typical clinical signs of colitis. Increased MPO activity confirmed the presence of inflammation. TNBS induced the development of megacolon, ulceration, transmural inflammatory infiltrate, and thickened bowel walls. Treatment with propolis moderately reduced the inflammatory response, decreased the number of cysts and abscesses, inhibited epithelial proliferation, and increased the number of goblet cells. The anti-inflammatory activity of the propolis SLNC 106 extract was confirmed by the reductions in both the inflammatory infiltrate and the number of cysts and abscesses in the colon mucosa.

## 1. Introduction

Crohn's disease (CD) and ulcerative colitis (UC) are chronic idiopathic inflammatory disorders that represent the two major types of inflammatory bowel disease (IBD). These diseases affect the gastrointestinal tract, and their course is characterized by alternating periods of remission and flare-up. The flare-up is manifested by abdominal pain, severe diarrhea, rectal bleeding, fever, weight loss, and potential systemic complications [1].

Although its cause is multifactorial, IBD depends on the presence of one or more genetically determined disorders that alter the barrier function of the bowel epithelium and lead to a greater exposure of the mucosal immune system to the normal components of the intestinal flora [2, 3].

The main therapeutic approach includes generic or selective anti-inflammatory agents and immunosuppressants. Treatment induces remission of the acute symptoms but is unable to cure the disease. In addition, 60 to 70% of the patients require surgical intervention due to complications [1].

Mesalazine (5-aminosalicylic acid) is one of the main agents used for flare-ups and to maintain remission in mild and moderate forms of UC and CD [1]. However, the biological activity of several other substances has been tested in recent decades [4–10]. Isolated propolis components, such as caffeic acid phenethyl ester (CAPE) and 3,5-diprenyl-4-hydroxycinnamic acid (artepillin C), and complete propolis extracts are promising alternatives because their biological activities (which include anti-inflammatory, antioxidant, immunomodulating, antimicrobial, and wound healing effects) are directly associated with treating inflammatory processes, such as IBD [11–16].

More than 300 substances have been identified as chemical components of propolis. The proportions of these compounds in propolis depend on the local flora [17, 18]. Phenolic compounds (flavonoids, aromatic acids, and benzopyrenes), di- and tri-terpenes, essential oils, aromatic acids and esters, aldehydes, ketones, and phenylpropanoids (caffeic and chlorogenic acids) are among the main components of propolis. Although the flavonoids [19] and phenolic acids are the components that are most directly related to tissue regeneration and to antimicrobial, antioxidative, and anti-inflammatory activities, the biological potential of propolis is probably a result of synergy between its components [17, 18] because the isolated compounds do not induce the same effects as the total extract [20, 21].

Due to its wide range of biological activities, particularly those that may be useful for treating IBD, the present study sought to assess the therapeutic effect of a hydroalcoholic propolis extract on experimentally induced colitis in rats. 

## 2. Materials and Methods

### 2.1. Animals

Fifty albino Wistar male adult rats (*Rattus norvegicus)* 90 days old and weighting 369.8 ± 26.25 g were obtained from the Central Biotery of the Universidade Estadual de Maringá (UEM), PR, Brazil. The animals were kept in polypropylene boxes (four animals per box) at a controlled temperature of 23 to 25°C and with a 12-hour light/dark cycle. The rats were fed standard rodent rations and water *ad libitum*. This study was approved by the Animal Experimentation Ethics Committee of UEM (Protocol 006/2008).

### 2.2. Propolis Extract Production

Propolis was produced in the apiary from Iguatemi Experimental Farm, UEM, Maringa, PR, Brazil, collected and stored at −22°C. The hydroalcoholic extract, prepared from the same batch, in order to avoid possible seasonal variations. The extract called SLNC106^(*PI*)^ (patent in progress), was performed in one step, and all variables were controlled and standardized in the laboratory of Phytotherapy Apitherapy and Development Maringá, PR, Brazil [22].

### 2.3. Experimental Procedure

Animals were fasted overnight with free access to water. Colitis was induced by a single enema instillation containing 0.8 mL of 20 mg of 2,4,6-trinitrobenzenesulfonic acid (TNBS) (Sigma Chemical Co., St. Louis, USA) in 50% ethanol [23]. 

The rats were treated with a 0.8 mL enema obtained by adding propolis extract to saline in the 8% (w/w) proportion or 0.8 mL of mesalazine solution (50 mg/Kg) (5-aminosalicylic acid-5-ASA) (EMS S/A, São Paulo, Brazil) as a reference drug [24]. Daily treatment started 48 hours after the colitis induction and lasted 5 or 12 days (ending at 7 or 14 days after the colitis induction), at which time the animals were euthanized. This delayed therapy approach was chosen to allow inflammation to develop [4]. The animals were randomly distributed among five groups of 10 animals each with *n* = 5 for each experimental time-point. Group S (control) received a single dose of 0.9% saline, group TNBS received TNBS solution, group SP received 0.9% saline and was treated with propolis, group TNBS-P received TBNS solution and was treated with propolis, and TNBS-M group received TNBS solution and were treated with mesalazine.

The following parameters were evaluated daily: body weight, vitality, stool appearance, and consistency. Numerical scores for stool consistency and rectal bleeding were calculated on a 0 to 2 scale: (0) stools with normal consistency; (1) liquid stools adhering to the anus without rectal bleeding; and (2) liquid stools and blood adhering to the anus (adapted from Lamprecht et al. 2001) [25]. Two hours before euthanasia, the animals received 0.5 mg/kg of intravenous (penian vein) vincristine sulfate (Tecnocris, Eurofarma, São Paulo, Brazil) as a mitotic blocker [26]. The animals were then euthanized with an overdose (40 mg/kg) of sodium thiopental. After laparotomy, the large intestine was removed, weighed, and measured. Distal colon samples were harvested for macroscopic assessment, measurement of myeloperoxidase activity, and histological processing for histomorphometric analysis.

### 2.4. Stereomicroscopy Assessment

Samples of the distal colon were opened by a longitudinal incision on the mesocolic margin, washed to remove feces, and observed under a stereomicroscope with trans-illumination (Olympus SZ 61, Tokyo, Japan). Alterations were scored on a 0 to 5 scale: (0) absence of inflamed areas; (1) localized hyperemia without ulcerations; (2) linear ulcers without significant inflammation and mild hyperemia; (3) ulcerations without necrosis (crusts) and 2 to 4 cm of inflammation; (4) ulcerations with crusts, megacolon, serosa adhering to organs, and 2 to 4 cm of inflammation; and (5) ulcerations with crusts, megacolon, serosa adherence involving several intestinal folds, stenosis and inflamed areas larger than 4 cm [23, 27]. 

### 2.5. Measurement of Myeloperoxidase (MPO) Activity

The progression of the inflammatory response was determined by measuring these myeloperoxidase (MPO) activity [28]. Samples from the distal colon samples were macerated and homogenized in a 50 mM pH 6.0 potassium phosphate buffer containing 0.5% (w/v) hexadecyltrimethylammonium bromide (HETAB) (Sigma Chemical Co., St. Louis, USA) with 50 mg of tissue in 1 mL of buffer solution. The samples were then subjected to an ultrasonic bath for 30 s, heated for two hours in a water bath at 60°C and centrifuged at 5000 g and 25°C for 10 minutes. Triplicate 10 *μ*L samples were removed from the supernatant and 200 *μ*L of the staining reagent was added; the staining reagent contained 4.2 mg of o-dianisidine dihydrochloride (Sigma Chemical Co., St. Louis, USA), 22.5 mL of double-distilled water, 2.5 mL of pH 6.0 50 mM potassium phosphate buffer, and 12.5 *μ*L of 1% hydrogen peroxide. The reaction was terminated after five minutes by adding 1.46 M sodium acetate, and the MPO activity was determined by the 450 nm absorbance, as measured by an ELISA reader (Lionheart Diagnostics, Status Labsystems, Multiskan RC, Uniscience, Brazil).

### 2.6. Histological Study

The samples from distal colon were washed with saline solution, fixed with Bouin's solution and embedded in paraffin. Semiserial 7 *μ*m sections perpendicular to the long axis of the colon were obtained. The hematoxylin-eosin (H&E)-stained sections were used for the following purposes: (1) morphometric analysis of the bowel layers; (b) inflammation assessment; (c) tissue damage assessment [29]; and (d) epithelial cell proliferation assessment using the metaphase index (MetI). The sections were stained using the Periodic Acid-Schiff (PAS) histochemical method to allow the goblet cells to be counted. The thickness of the bowel mucosa, submucosa, muscular and serosa layers and complete intestinal wall was assessed under 4x magnification at 10 random sites from the histological sections (50 measures/animal/layer). The number of goblet cells was determined by counting 100 microscopic fields/animal over a total area of 9.06 mm^2^ at 40x magnification.The morphometric analysis and goblet cell counting were performed with images obtained from a QColor 3 camera (Olympus American INC, Canada) coupled to an Olympus BX 41 optic trinocular microscope (Tokyo, Japan). The bowel layers were measured using Image-Pro plus 4.5 image analysis software (Media Cybernetics, Silver Spring, MD). The microscopic inflammation and tissue damage were scored on a 0 to 3 severity scale using the following criteria: (a) ulceration, (b) abscesses in crypts, (c) cysts, (d) damaged wall architecture, (e) inflammatory infiltrates, and (f) vascular dilatation; a detailed description is given in Fabia et al., 1993 [29]. The metaphasic index (MetI), the ratio of the nuclei in metaphase to the total number of counted nuclei, was determined for the longitudinal crypts that exhibited evident lumens; 2.500 cells were counted for each animal [30] using an Olympus BX41 optic binocular microscope (Tokyo, Japan) with 40x magnification. The MetI was multiplied by Tannock's factor to correct tissue geometry, thereby avoiding overestimates of the number of metaphase nuclei [31].

### 2.7. Statistical Analysis

The Kruskal-Wallis test followed by Dunn's posttest were used for the nonparametric numerical variables. The means of the parametric variables were compared using one- and two-way variance analysis (ANOVA) models and Tukey or Bonferroni posttest. The statistical analysis was performed using GraphPad Prism 5.0 software (GraphPad Software, Inc. San Diego, CA, USA), and the results are expressed as mean ± standard deviation. Significance was established at 5%.

## 3. Results

### 3.1. Clinical Assessment

The animals in the TNBS, TNBS-M, and TNBS-P groups exhibited reduced vitality and piloerection and 100% developed severe diarrhea one day after the colitis was induced. Administering saline or saline + propolis did not result in clinical alterations, but the animals remained active, and the consistency and appearance of the stools were normal.  Table 1 describes the stool appearance and consistency scores.

**Figure 1:**
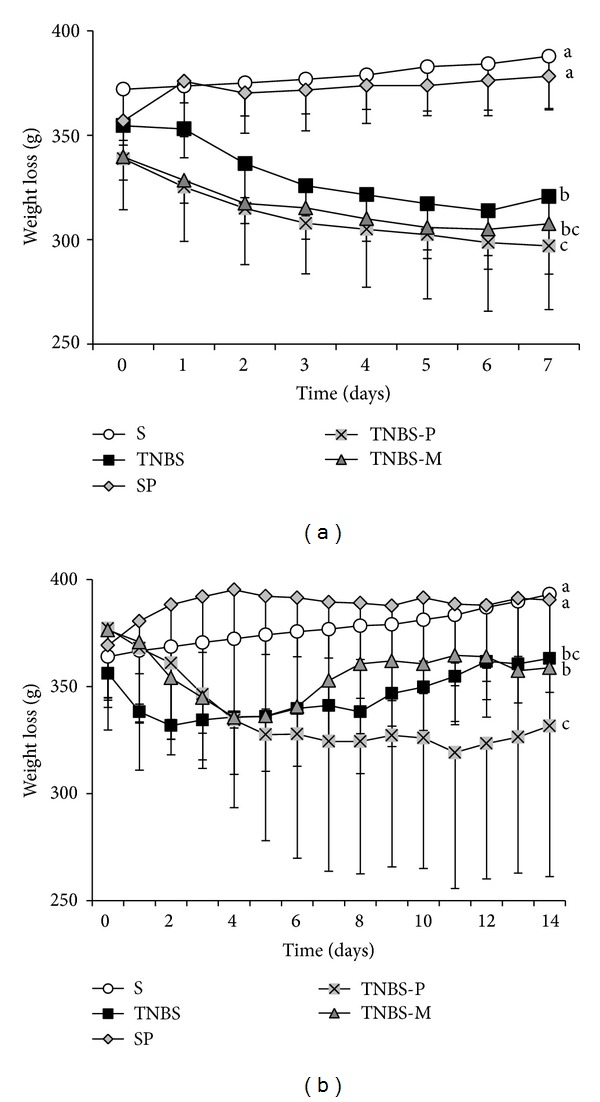
The body weight of rats that received rectal TNBS (to induce colitis) or saline solution (S) after treatment for 5 or 12 days with propolis hydroalcoholic solution (TNBS-P and SP) or mesalazine solution (TNBS-M). (a) The animals euthanized 7 days after the colitis was induced. (b) The animals euthanized 14 days after the colitis was induced. Different letters indicate significant differences in a one-way ANOVA model with Tukey's post-test (*P* < 0.05) (*n* = 5).

Throughout the study, the animals in the TNBS, TNBS-P and TNBS-M groups had a significantly greater weight loss (*P* < 0.05) than that of the animals in the control groups (Figure 1). Body weight recovery was at its lowest in the group treated with propolis solution.

### 3.2. Stereomicroscopy Analysis

The results of the stereomicroscopic analysis are described in Table 2. The animals in group S exhibited no alterations. Approximately 60% of the animals in group SP exhibited hyperemic areas after five days of treatment, and 20% had hyperemia after 12 days. One week after the colitis was induced, groups TNBS and TNBS-M exhibited tissue lesions and had scores significantly different from those of group S. Similar but milder lesions were present in group TNBS-P. Although there were lesions with differing degrees of severity (ranging from hyperemic areas in the mucosa to healed ulcers exhibiting whitish devitalized tissue) in the groups with induced colitis (independent of treatment), the differences in the lesions were not statistically significant 12 days after the onset of treatment. 

### 3.3. Width and Weight/Length Ratios

The distal colon width and the large intestine weight/length ratios indicated edema and are described in Table 3.

### 3.4. Measurement of Myeloperoxidase (MPO)

The MPO activity is described in Table 4. The MPO activity was significantly greater (*P* < 0.05) in groups TNBS, TNBS-P, and TNBS-M than in groups S and SP at 7 and 14 days. The MPO activity was higher in the animals treated with propolis than in the animals with untreated colitis at 14 days (Table 4).

### 3.5. Histomorphometric Assessment

#### 3.5.1. Microscopic Analysis

**Figure 2:**
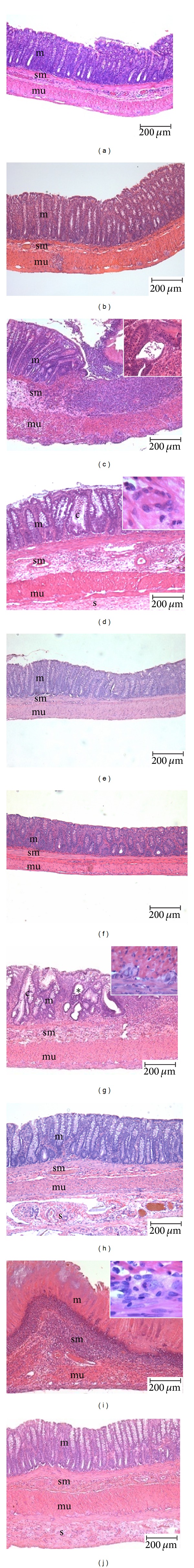
Photomicrography of the distal colons of rats that received rectal TNBS (to induce colitis) or saline solution (S) after treatment for 5 or 12 days with propolis hydroalcoholic solution (TNBS-P and SP) or mesalazine solution (TNBS-M). The animals were euthanized 7 or 14 days after the colitis was induced. The animals in groups S ((a) and (b)) and SP ((e) and (f)) exhibited normal histological characteristics (m: mucosa, sm: submucosa, and mu: muscular). Group TNBS ((c) and (d)). (c) shows extensive ulceration and intense inflammatory infiltrate surrounded by normal mucosa; a crypt abscess containing polymorphonuclear cells is shown in detail. In (d), note the distorted crypts (c), presence of foreign body giant cells in the submucosa (detail), and expansion of the serosa (s). The group TNBS-P ((g) and (h)) mucosa shows regeneration with bifurcated crypts (c) and cysts (∗) in (g) and serosa expansion (S) in (h). Group TNBS-M ((i) and (j)). In (i), note the ulcerated area in the mucosa and intense transmural inflammatory infiltrate. In the detail, note the presence of eosinophils inside a myenteric ganglion. In (j), note the restored mucosa, absence of inflammatory infiltrate, and expanded serosa (s). Hematoxylin-eosin. Scale = 200 *μ*m.

The animals in groups S and SP exhibited normal histological characteristics in the distal colon and had lymphatic nodules in the submucosa (Figures 2(a), 2(b), 2(e) and 2(f)). The distal colons of the animals with colitis exhibited similar characteristics after the TNBS was administered, regardless of the treatment. Tissue damage and loss of superficial cells were observed on day 7, resulting in multiple ulcerations and dense inflammatory infiltrates with a predominance of neutrophils and eosinophils. A large number of crypts were deformed by cysts or microabscesses and exhibited neutrophilic exudates. In addition to edema, these exudates also caused the loss of crypt epithelium and goblet cells (Figures 2(c) and 2(i)). The areas of regenerated mucosa exhibited bifurcated crypts with broadened bases, wide lumens, irregular shapes, cysts and abscesses. The submucosa contained dense transmural inflammatory infiltrates that consisted predominantly of neutrophils (with some eosinophils, plasmocytes, macrophages, and phagocytic epithelial cells), whereas only polymorphonuclear cells were observed in the lymph nodes of the submucosa and myenteric plexuses (Figures 2(d), 2(g), and 2(h)). An expanded serosa with adipose tissue exhibiting a lymphocyte and macrophage infiltrates and increased vascularization was also observed (Figures 2(h) and 2(j)). The distal colons of the rats in the TNBS, TNBS-P, and TNBS-M groups exhibited characteristics on day 14 that were similar to those observed on day 7, although milder.

**Figure 3:**
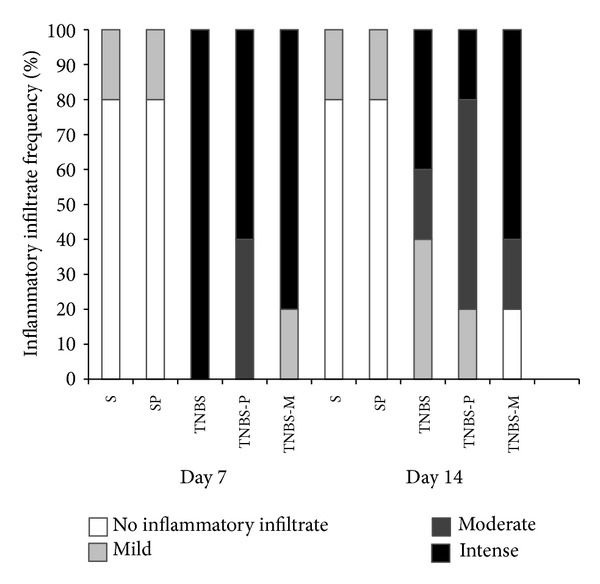
Frequency with which animals exhibited (a) intense, moderate, mild, or no inflammatory infiltrate after they received rectal TNBS (to induce colitis) or saline solution (S) and were treated for 5 or 12 days with propolis hydroalcoholic solution.

**Figure 4:**
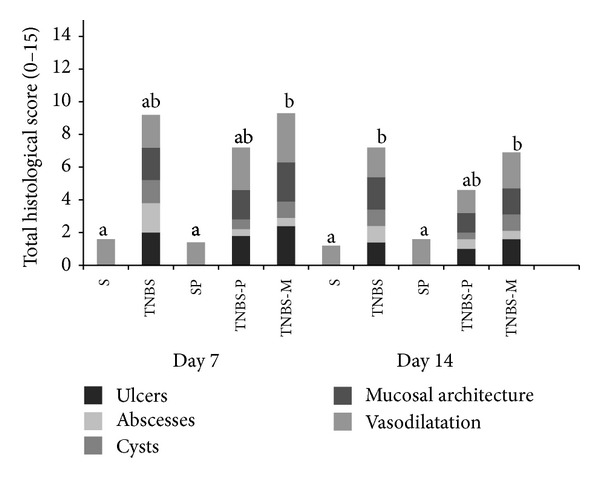
The total score of animals exhibiting histological changes (alterations in the architecture of the distal colon wall, cysts, abscesses, and ulcers) after they received rectal TNBS (to induce colitis) or saline solution (S) and were treated for 5 or 12 days with propolis hydroalcoholic solution (TNBS-P) or mesalazine solution (TNBS-M). Means ± SD followed by different letters on the day of the animals' deaths indicate a significant difference according to the Kruskal-Wallis test and Dunn's post-test (*P* < 0.05); (*n* = 5).

Figure 3 describes the frequencies at which the animals exhibited dense, moderate, mild, or no inflammatory infiltrate, and Figure 4 describes the frequency of the assessed histological variables: colon wall architecture, presence of cysts, abscesses, and ulcers.

#### 3.5.2. Morphometry of the Bowel Layers

The thickness of the bowel layers and complete distal colon wall is described in Table 5.

#### 3.5.3. Number of Goblet Cells

**Figure 5:**
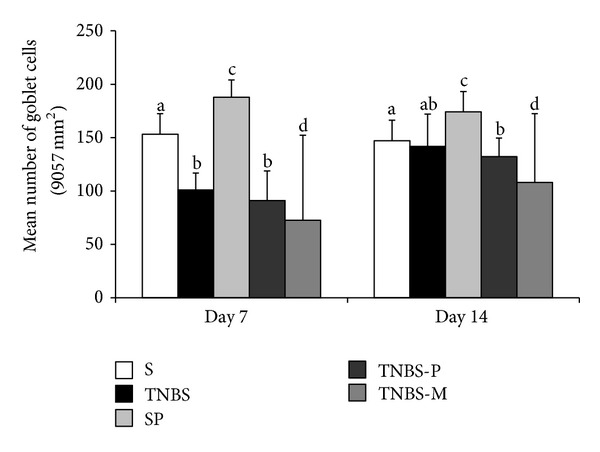
The number of goblet cells in the distal colons of rats (area of 9.06 mm^2^ at 40x magnification) that received rectal TNBS (to induce colitis) or saline solution (S) and were treated for 5 or 12 days with propolis hydroalcoholic solution (TNBS-P) or mesalazine solution (TNBS-M). The animals were euthanized on day 7 (a) or 14 (b) after the colitis was induced. Means ± SD followed by different letters on the day of the animals' deaths indicate a significant difference according to the Kruskal-Wallis test and Dunn's post-test (*P* < 0.05), (*n* = 5).

The number of goblet cells in the intestinal glands of the animals with induced colitis decreased. This decrease was more pronounced 7 days after induction. The animals in the TNBS-M group had the fewest goblet cells on day 7 (Figure 5(a)). By contrast, the number of goblet cells increased (*P* < 0.05) in the healthy animals that were administered the propolis solution (group SP). Similar results were observed on day 14, except that the TNBS group had goblet cells populations that were similar to those of the untreated control animals (group S) (Figure 5(b)).

#### 3.5.4. Assessment of Epithelial Cells

Table 6 describes the epithelial proliferation results. Colitis treatments using the mesalazine (at 7 and 14 days) or propolis solutions (14 days) inhibited intestinal gland cell proliferation.

## 4. Discussion

We previously demonstrated the wound healing effect, effectiveness of anti-inflammatory, and antimicrobial of propolis ethanolic extracts [16, 32, 33], and therefore the interest in evaluating their activity in a model of experimental colitis.

Colitis was induced in rats by rectally administering a single dose of 20 mg TNBS in 50% (v/v) ethanol in the distal colon and was assessed for 14 days. The therapeutic effect of a hydroalcoholic propolis (SLNC 106) solution was assessed and compared to that of the standard medication, namely, mesalazine. 

In the present study, all of the animals that received TNBS exhibited the typical clinical signs of colitis after 24 hours: piloerection, hypoactivity, weight loss, and diarrhea. These signs improved after 48 hours. Severe diarrhea with rectal bleeding is characteristic of inflammatory bowel disease (IBD). Severe diarrhea is the result of functional and structural alterations in the gastrointestinal tract and is associated with inflammation, nausea, and abdominal pain, which occur mainly in the active disease phase [34, 35]. 

The inflammatory status of the distal colon was biochemically characterized by myeloperoxidase (MPO) enzyme activity and morphologically characterized by a series of indicators, including hyperemia, ulcerations, width and weight/length ratios of the distal colon, and histomorphometric assessment of the intestinal wall.

From the macroscopic perspective, megacolon was observed in all of the animals with colitis. However, there were no differences in bowel width among the treated and control animals, suggesting that propolis and mesalazine may have attenuated the development of megacolon during the first week. During the second week, the animals in the TNBS group exhibited a decrease in distal colon width that was similar to that of the treated animals, even though they did not receive treatment; therefore, this parameter improves over time, regardless of pharmacological treatment. A degree of adherence to adjacent organs and mesenteric fat accumulation was observed at 7 and 14 days in a strong contrast to the appearance of the colon in the healthy animals.

In this experimental colitis model, megacolon develops as a consequence of dysmotility arising from changes in the structure and function of the enteric nervous system (ENS) [34]. TNBS-induced colitis is associated with a 20% loss of myenteric neurons, which occurs when neutrophils infiltrate the ganglia. Interestingly, this neuronal loss seems to persist for a period of time that is coincident with the resolution of inflammation. The reduction in myenteric neurons has not been associated with any particular subpopulation, which suggests that indiscriminate loss occurs at the onset of TNBS-induced colitis [36]. 

Inflammation is known to affect bowel function. In the present study, MPO activity remained high throughout the assessed period. Neither the propolis extract nor the mesalazine were able to reduce this enzyme activity, which was higher (*P* < 0.05) in the animals treated with propolis than in those treated with mesalazine. This difference may suggest aggravation of the inflammatory process, particularly in the animals treated with propolis; however, the anti-inflammatory effect of propolis was apparent after five days of administration when 40% of the animals already exhibited moderate inflammatory infiltrate. At five days, only 20% of the animals treated with mesalazine exhibited decreased inflammation, and any decreases were classified as mild.

Twelve days after the propolis administration, inflammation was reduced in 80% of the animals, 60% had moderate infiltrates and 20% had mild infiltrates. At this time, 60% of the animals treated with mesalazine still had dense infiltrates, 20% had mild infiltrates, and 20% no longer exhibited inflammation.

These results suggest that the therapeutic effect of the propolis treatment was modulated, that is, its action was slower, but its scope was wider because it encompassed a higher number of animals than did the mesalazine treatment. The anti-inflammatory effect of mesalazine was more rapid and more pronounced only in the animals that responded well to it (less than half of the treated population). The modulated anti-inflammatory action of propolis and the restricted action of mesalazine may explain the high MPO activity levels that were observed. The inflammation persisted after 12 days of treatment with both drugs.

All of the animals with colitis that did not receive pharmacological treatments exhibited dense inflammatory infiltrate in the first week. On day 14, the inflammation had spontaneously decreased in most (60%) of the animals (20% had moderate and 40% had mild infiltrate), whereas 40% still exhibited dense infiltrate. 

The combined and separate therapeutic effects of propolis and mesalazine in an acetic acid-induced colitis model were investigated. Most of the animals treated with propolis exhibited normal histology, and 50% and 33% of the rats treated with mesalazine alone or in combination with propolis, respectively, exhibited inflammatory infiltration. The authors concluded that the drugs are effective both alone and in combination but that the combined effect was not cumulative for experimental colitis [37]. 

The anti-inflammatory effect of propolis has been attributed to several active components: caffeic acid, quercetin, naringenin, CAPE [38], coumaric acid, ferulic acid, campherol, and galangin [39]. The possible mechanisms include prostaglandin suppression, leukotriene synthesis by macrophages, and inhibition of the myeloperoxidase, NADPH-oxidase, ornithine decarboxylase, and protein tyrosine kinase enzymes [40]. Propolis also stimulates macrophages and phagocytic activities [41]. 

Phenolic compounds, particularly flavonoids, are thought to remove the excess free radicals produced by inflammation. Although oxidative damage is already known to be associated with tissue destruction, modulating free radical production may represent a new direction in IBD treatment [41]. 

CAPE is an important antioxidant that also has anti-inflammatory properties because it inhibits arachidonic acid release from the cell membrane and thus suppresses cyclooxygenase (COX-1 and COX-2) activity [42]. It is also a powerful and specific inhibitor of nuclear factor-kappa B (NF-kB) [43], which is super-expressed in the lamina propria of Crohn's disease patients [3] and is a pathogenic factor in TNBS-induced colitis models [44]. 

The NF-kB is activated by several factors associated with IBD, such as inflammatory cytokines (interleukin-1 (IL-1), tumor necrosis factor alpha (TNF-*α*)), bacterial products and oxidative stress. The NF-kB controls IL-1*β* expression and inducible nitric oxide synthase (iNOS) in macrophages. The corresponding mRNA expression is suppressed at the transcriptional level after an ethanolic extract of propolis is added to a J774A macrophage cell culture line [45], suggesting that propolis or its components inhibit the inflammatory response via different mechanisms. Allied to this, it was found that TNBS-induced colitis in mice previously fed (14 days before induction of colitis) with standard food containing propolis extract showed an improvement of colitis symptoms in a dose-dependent manner by inhibiting the Th1 cells differentiations [46].

The histological analysis in the present study revealed a predominance of neutrophils in the early acute phase that was followed by eosinophilic domination of the transmural infiltrate. Polymorphonuclear cells were found near or even inside the myenteric ganglia and may be associated with enteric nervous system effects and neuronal death [47, 48].

Transmural inflammation may result in fistulas, abscesses, and fissures due to serosal infiltration of adjacent loops. Such an infiltration results in fibrin deposits and makes the mesenteric surfaces of the inflamed loops adherent between themselves and to other abdominal organs, thereby favoring the formation of fistulas and fibrous adherences [49]. 

Experimental data suggest that even moderate inflammation may cause persistent alterations in the nervous function and smooth muscle of the gastrointestinal tract. These alterations can result in colon dysmotility, hypersensitivity, and dysfunction, even when the inflammation is restricted to the proximal small intestine. Alterations in bowel function are observed after resolution of acute intestinal inflammation, suggesting that the changes it induces remain after recovery, and play an important role in producing IBD symptoms [50]. 

Macroscopically assessing colitis lesions underestimates their intensity; therefore, it should complement rather than replace microscopic analysis. Therefore, the present study microscopically assessed the frequency of the following histological variables: cysts, abscesses, ulcers, and alterations in the structure of the intestinal wall.

Crypt abscesses when totally developed, characteristically show neutrophils inside, in the crypt wall and adjacent lamina propria. In addition to edema, this exudate may also cause a loss of the crypt epithelium and goblet cells [51]. By contrast, cysts typically do not contain inflammatory cells inside. The propolis treatment was beneficial because it decreased the number of abscesses and cysts.

The hyperemia and ulcerations analyses determine the commitment of tissues. All of the induced colitis groups exhibited a range of alterations, from hyperemia to ulcerations, with various degrees of severity. The propolis and mesalazine treatments caused a small decrease in the lesion score only after 12 days of treatment, suggesting that neither of the drugs were able to reduce hyperemia and/or ulceration.

An increase in the weight/length ratio is a frequent finding in TNBS-induced colitis [9–11] and serves as an indicator of edema. In the present study, this ratio remained significantly higher in animals with colitis at days 7 and 14, indicating a persistent inflammatory state. This finding is consistent with other studies in which treatment also failed to reduce the colon weight increase and the weight/length ratio [8, 10]. The injuries caused by TNBS can sometimes reach a magnitude that is difficult to overcome by pharmacological treatment, which may explain why these treatments do not affect certain parameters [8]. 

All of the animals with colitis exhibited a thickening of the distal colon walls, mostly at the submucosa and serosa. In addition to the inflammatory infiltrate, the submucosa exhibited dilated vessels and edema, which explains its expansion. The thickening of the serosa was characterized by expansion, extensive vascularization, and infiltration by immune cells. A remarkable pathological angiogenesis, in which vessels originating in the submucosa penetrate the muscular layer towards the serosa, is associated with the chronic stages of inflammatory bowel disease [52].

In addition, the muscular layer increased significantly during the first week; this process is usually, partially, attributed to the accumulated inflammatory cells [23], inflammatory hypertrophy and hyperplasia of the muscular cells, and altered protein content. These factors explain the intestinal motility disorders observed in TNBS-induced inflammation models [53]. These alterations resulted in a notable increase in total wall thickness mainly on day 7. An increased total intestinal wall thickness is typical of this colitis model [23] and is probably associated with the inflammatory factors mentioned above.

Inflammation may interfere with the migration and proliferation of epithelial cells and thus modulates bowel epithelium repair [54]. Treatment with propolis and mesalazine reduced proliferative activity in the crypts relative to the TNBS group. The inhibitory effect of the propolis treatment was more noticeable after 12 days, whereas the effect inhibitory of the mesalazine was already perceptible on day 5. The untreated animals with colitis exhibited the highest proliferation rates. 

The metaphasic index did not differ between the TNBS and control S groups; only the TNBS-P and TNBS-M groups differed significantly from that of the TNBS group, suggesting that the metaphasic index was increased (although not significantly) in the animals with untreated colitis. This hypothesis is strengthened by the presence of bifurcated crypts in the TNBS group, which suggests that a regenerative response may have been triggered by TNBS-induced epithelial erosion stimulating reproduction by the budding or branching of new crypts from surviving crypts. This phenomenon is a signature of the hyperplasic response and includes a reduction in the duration of the cell cycle and an expansion of the proliferative compartment [30]. The animals in the control group that were administered the propolis solution exhibited low proliferation indices, which suggests an inhibitory effect of propolis that is independent of inflammation condition. 

Cell proliferation in experimental colitis models is controversial. Both, increased [55, 56] and decreased [57] proliferative indices have been reported.

The function of other epithelial cells may also be affected. Thus, fewer goblet cells were found in the animals with colitis during the first week, independent of treatment. With the exception of the group treated with mesalazine, the epithelial cell population recovered. In the controls, the propolis exhibited a stimulatory effect on goblet cells. Several studies have reported a remarkable reduction in goblets cells, more severe colitis, and reduced mucin content after TNBS-induced inflammation [4, 48, 58]. By contrast, Torres et al. (1999) [55] found an increase in the number and size of goblet cells and suggested that this phenomenon is characteristic of TNBS-induced cryptitis; the increased cellularity may reflect the increased proliferation as a tissue repair mechanism in response to TNBS.

In general, the literature suggests that experimental colitis treatments are more effective when applied preventively before colitis is induced [4, 9]. In the present study, treatment was initiated 48 hours after induction (i.e., when the inflammatory process was already established) and it was, thus, a late therapeutic approach [4]. In this context, we concluded that the therapeutic anti-inflammatory potential of the propolis hydroalcoholic extract could be established by the decreased intensity of the inflammatory infiltrate and by the reduction in the number of cysts and abscesses in the colonic mucosa. 

## Figures and Tables

**Table 1:** The characteristics of stools from rats that received rectal TNBS (to induce colitis) or saline solution (S) after 5 or 12 days of treatment with propolis hydroalcoholic solution (TNBS-P and SP) or mesalazine solution (TNBS-M). The animals were euthanized 7 or 14 days after the colitis was induced. The results are expressed as the mean ± standard deviation (*n* = 5).

Group/time (days)	Characteristics of stools (0–2) (0) Well-formed pellets (1) Liquid stools stuck to the anus, no bleeding (2) Liquid stools and blood stuck to the anus	
	Day 7	Day 14

S	0^a^	0^a^
TNBS	2.0 ± 0^b^	1.6 ± 0.55^b^
SP	0^a^	0^a^
TNBS-P	1.6 ± 0.55^b^	1.0 ± 0^ab^
TNBS-M	1.0 ± 0^ab^	1.0 ± 0^ab^

^a,b^Means followed by different letters in the same column are significantly different according to the Kruskal-Wallis test and Dunn's post-test (*P* < 0.05).

**Table 2:** Stereomicroscopic assessment of the distal colons of rats that received rectal TNBS (to induce colitis) or saline solution (S) after treatment for 5 or 12 days with propolis hydroalcoholic solution (TNBS-P and SP) or mesalazine solution (TNBS-M). The animals were euthanized 7 or 14 days after the colitis was induced. The results are expressed as the mean ± standard deviation (*n* = 5).

Group/time (days)	Score (0–5) (0) Absence of inflamed areas (1) Localized hyperemia without ulcerations (2) Mild hyperemia, linear ulcers without significant inflammation (3) Ulcerations without necrosis (crusts), 2 to 4 cm of inflammation (4) Ulcerations with crusts, 2 to 4 cm of inflammation (5) Ulcerations with crusts (inflammation >4 cm)	
	Day 7	Day 14

S	0^a^	0^a^
TNBS	4.6 ± 0.55^b^	3.2 ± 1.79^b^
SP	0.6 ± 0.55^a^	0.2 ± 0.45^a^
TNBS-P	3.6 ± 1.14^ab^	3.0 ± 2.0^ab^
TNBS-M	4.2 ± 1.30^b^	3.8 ± 1.79^b^

^a,b^The means followed by different letters in the same column are significantly different according to the Kruskal-Wallis test and Dunn's post-test (*P* < 0.05).

**Table 3:** The width and weight/length ratio (mg/cm) of the distal colons of rats that received rectal TNBS (to induce colitis) or saline solution (S) after treatment for 5 or 12 days with propolis hydroalcoholic solution (TNBS-P and SP) or mesalazine solution (TNBS-M). The animals were euthanized 7 or 14 days after the colitis was induced. The results are expressed as the mean ± standard deviation (*n* = 5).

Group/time (days)	Width (cm)		Weight/length ratio (mg/cm)	
	Day 7	Day 14	Day 7	Day 14
S	1.02 ± 0.18^a^	1.00 ± 0.07^a^	252.4 ± 61.1^a^	268.0 ± 42.6^a^
TNBS	1.56 ± 0.31^b^	1.36 ± 0.15^a^	558.2 ± 46.4^b^	415.1 ± 105.9^a^
SP	1.02 ± 0.18^a^	1.04 ± 0.19^a^	236.7 ± 41.6^a^	259.8 ± 18.0^a^
TNBS-P	1.26 ± 0.18^a^	1.25 ± 0.18^a^	500.7 ± 125.3^bc^	687.3 ± 169.0^c^
TNBS-M	1.16 ± 0.15^a^	1.25 ± 0.11^a^	423.2 ± 90.6^c^	605.9 ± 67.1^c^

^a,b,c^The means followed by different letters in the same column are significantly different according to a one-way ANOVA model and Tukey's post-test (*P* < 0.05).

**Table 4:** The myeloperoxidase activity (MPO) (nm) in the distal colons of rats that received rectal TNBS (to induce colitis) or saline solution (S) after treatment for 5 or 12 days with propolis hydroalcoholic solution (TNBS-P and SP) or mesalazine solution (TNBS-M). The animals were euthanized 7 or 14 days after the colitis was induced. The results are expressed as the mean ± standard deviation (*n* = 5).

MPO activity (OD 450 nm)		
Group/time (days)	Day 7	Day 14
S	0.185 ± 0.041^a^	0.166 ± 0.071^a^
TNBS	0.618 ± 0.161^b^	0.659 ± 0.124^b^
SP	0.068 ± 0.074^a^	0.180 ± 0.097^a^
TNBS-P	0.720 ± 0.051^b^	0.823 ± 0.029^c^
TNBS-M	0.749 ± 0.172^b^	0.782 ± 0.046^bc^

^a,b,c^The means followed by different letters in the same column are significantly different according a to two-way ANOVA model and the Bonferroni post-test (*P* < 0.05).

**Table 5:** A morphometric analysis (by group and time) of the bowel layers and complete wall in the distal colons of rats that received rectal TNBS (20 mg/50% ethanol) (TNBS, TNBS-P, TNBS-M) or saline solution (S and SP) and were rectally treated with mesalazine (TNBS-M) or propolis SLNC106 (SP and TNBS-P). The results are expressed as the mean ± standard deviation (*n* = 5).

Group/time	Mucosa (*μ*m)	Submucosa (*μ*m)	Muscle layer (*μ*m)	Serosa (*μ*m)	Total wall (*μ*m)
Day 7					
S	308.7 ± 32.2^a^	70.1 ± 21.9^a^	193.9 ± 14.9^a^	16.0 ± 1.4^a^	563.9 ± 69.6^a^
TNBS	388.9 ± 75.2^ab^	382.2 ± 158.1^b^	257.6 ± 81.4^a^	35.9 ± 24.3^a^	1062.0 ± 265.8^b^
SP	325.4 ± 20.9^a^	59.9 ± 9.3^a^	187.8 ± 45.4^a^	25.2 ± 7.6^a^	580.3 ± 50.2^a^
TNBS-P	447.7 ± 81.5^b^	241.1 ± 148.6^ab^	318.4 ± 76.2^ab^	82.4 ± 74.7^a^	1033.0 ± 119.2^b^
TNBS-M	456.6 ± 60.3^b^	271.4 ± 204.4^ab^	470.8 ± 350.0^b^	192.8 ± 153.3^b^	1320.0 ± 650.1^b^
Day 14					
S	347.8 ± 30.6^a^	82.11 ± 27.7^a^	250.4 ± 51.9^a^	16.4 ± 1.5^a^	677.8 ± 30.9^a^
TNBS	362.0 ± 44.5^a^	235.2 ± 101.5^a^	200.5 ± 57.4^a^	73.9 ± 21.6^ab^	888.1 ± 174.3^a^
SP	321.1 ± 5.6^a^	126.9 ± 118.8^a^	177.0 ± 55.7^a^	21.0 ± 6.9^a^	675.0 ± 302.9^a^
TNBS-P	460.3 ± 50.7^b^	257.9 ± 203.5^a^	282.3 ± 84.8^a^	132.7 ± 57.9^b^	1121.0 ± 252.1^b^
TNBS-M	464.8 ± 100.4^b^	270.6 ± 204.3^a^	342.0 ± 84.0^a^	250.4 ± 111.4^c^	1312.0 ± 391.7^b^

^a,b,c^The means followed by different lowercase letters in the same column were significantly different at 7 and 14 days according to a two-way ANOVA model and the Bonferroni post-test (*P* < 0.05).

**Table 6:** The metaphase index (MetI) in the epithelium of the distal colons of rats that received rectal TNBS (to induce colitis) or saline solution (S) after treatment for 5 or 12 days with propolis hydroalcoholic solution (TNBS-P and SP) or mesalazine.

Group/time	Metaphase Index (MetI) (%)	
	Day 7	Day 14
S	6.945 ± 0.8575	8.872 ± 0.7792
TNBS	9.099 ± 2.722	10.51 ± 0.8000
SP	6.373 ± 1.042	5.68 ± 0.827
TNBS-P	7.468 ± 2.029	6.038 ± 1.102^a^
TNBS-M	4.528 ± 3.298^a^	5.663 ± 1.053^a^

^a^ *P* < 0.05 compared to TNBS in the same column through a one-way ANOVA model and Tukey's post-test (mean ± standard deviation, *n* = 4).
